# Elevated mean platelet volume predicts poor prognosis in colorectal cancer

**DOI:** 10.1038/s41598-017-11053-y

**Published:** 2017-08-31

**Authors:** Na Li, Zhiwei Yu, Xin Zhang, Tiemin Liu, Yu-xiang Sun, Rui-tao Wang, Kai-jiang Yu

**Affiliations:** 1 Department of Internal Medicine, Harbin Medical University Cancer Hospital, Harbin Medical University, Harbin, Heilongjiang 150081 China; 2 Department of Colorectal Surgery, Harbin Medical University Cancer Hospital, Harbin Medical University, Harbin, Heilongjiang 150081 China; 3 Division of Hypothalamic Research, Department of Internal Medicine, UT Southwestern Medical Center, Dallas, TX 75390 USA; 4 Children’s Nutrition Research Center, Huffington Center on Aging, Departments of Pediatrics & Molecular and Cellular Biology, Baylor College of Medicine, Houston, Texas 77030 USA; 5 Department of Intensive Care Unit, Harbin Medical University Cancer Hospital, Harbin Medical University, Harbin, Heilongjiang 150081 China; 6 Heilongjiang Academy of Medical Science, Harbin, Heilongjiang 150081 China

## Abstract

Altered mean platelet volume (MPV) is implicated in several malignancies. However, the clinicopathological significance and prognostic value of MPV in colorectal cancer (CRC) is still elusive. The purpose of this study was to elucidate the predictive significance of MPV in CRC. The retrospective study recruited 509 consecutive CRC patients between January 2009 and December 2009. The relationships between MPV and clinicopathological characteristics were analyzed. Kaplan-Meier method and Cox regression were used to evaluate the prognostic impact of MPV. Of the 509 CRC patients, high MPV levels were detected in 150 (29.5%) patients. Elevated MPV was associated with tumor differentiation (p < 0.001). Patients with increased MPV had poor overall survival compared with those with normal level (60.0% vs. 83.6%, log-rank test, p = 0.035). Cox regression analysis showed that MPV was an independent prognostic factor in CRC (HR = 1.452, 95% CI = 1.118–1.884, p = 0.005). In conclusion, MPV is easily available in routine blood test. Elevated MPV might act as a marker of prognosis and therapeutic target for CRC.

## Introduction

Colorectal cancer (CRC) is the third most commonly diagnosed cancer in males and the second in females. Although there are many established therapeutic strategies including surgery, chemotherapy and radiotherapy, the high rate of relapse and distant metastasis still threaten a large percentage of patients. Therefore, identification of appropriate markers for prognosis in CRC is of great importance.

Platelets play a pivotal role in cancer progression and metastasis. There is emerging evidence to suggest that platelets mediate tumor cell growth, angiogenesis, and dissemination^1^. Increased platelets are correlated with a decrease in overall survival and poorer prognosis in various types of cancer, including pancreatic cancer, gastric cancer, colorectal cancer, endometrial cancer, and ovarian cancer^2–6^. However, platelet count is determined by the balance between the rate of production and consumption of platelets. A normal platelet count could conceal the presence of highly hypercoagulative and pro-inflammatory cancer phenotypes in the presence of efficient compensatory mechanisms^7^.

Mean platelet volume (MPV), the most commonly used measure of platelet size, is a surrogate marker of platelet activation^8^. Altered MPV levels were found in gastric cancer, ovarian cancer, lung cancer, and breast cancer^9–12^. Our previous study demonstrated that MPV was elevated in CRC^13^. In a small-size sample study, MPV was found to be a prognostic indicator in metastatic colorectal cancer patients treated with bevacizumab-combined chemotherapy^14^.

In the present study, we investigated the effect of MPV levels on pathological parameters and clinical outcome.

## Results

Between Jan, 2009 and Dec, 2009, a total of 509 CRC patients were enrolled in this study. Among the 509 patients, 239 (47.0) were women and 270 (53.0) were men, and the median age was 58.1 ± 9.9 years (range 30–87). The characteristics of the patients are reported in Table 1. In terms of the staging system, 243 cases were categorized as stage I and stage II, 266 as stage III and stage IV.

**Table 1:** Baseline characteristics of patients with colorectal cancer according to MPV levels.

Variables	Total n (%)	MPV ≤ 8.6 n (%)	MPV > 8.6 n (%)	P value
Age (years)				0.162
<65	372 (73.1)	256 (71.3)	116 (77.3)	
≥65	137 (26.9)	103 (28.7)	34 (22.7)	
Gender				0.278
Male	270 (53.0)	196 (54.6)	74 (49.3)	
Female	239 (47.0)	163 (45.4)	76 (50.7)	
Tumor location				0.812
Colon	279 (54.8)	198 (55.2)	81 (54.0)	
Rectum	230 (45.2)	161 (44.8)	69 (46.0)	
Tumor size (cm)				0.276
<5.0	327 (64.2)	236 (65.7)	91 (60.7)	
≥5.0	182 (35.8)	123 (34.3)	59 (39.3)	
Differentiation				<0.001
Well	47 (9.2)	32 (8.9)	15 (10.0)	
Moderate	321 (63.1)	229 (63.8)	92 (61.3)	
Poor	141 (27.7)	98 (27.3)	43 (28.7)	
T classification				0.593
T1 + T2	85 (16.7)	62 (17.3)	23 (15.3)	
T3 + T4	424 (83.3)	297 (82.7)	127 (84.7)	
Lymph node metastasis				0.823
Absent	254 (49.9)	178 (49.6)	76 (50.7)	
Present	255 (50.1)	181 (50.4)	74 (49.3)	
Distant metastasis				0.496
Absent	465 (91.4)	326 (90.8)	139 (92.7)	
Present	44 (8.6)	33 (9.2)	11 (7.3)	
TNM stage				0.787
I/II	243 (47.7)	170 (47.4)	73 (48.7)	
III/IV	266 (52.3)	189 (52.6)	77 (51.3)	
Adjuvant therapy				0.271
Yes	438(86.1)	305 (85.0)	133(88.7)	
No	71 (13.9)	54 (15.0)	17 (11.3)	

**Figure 1:**
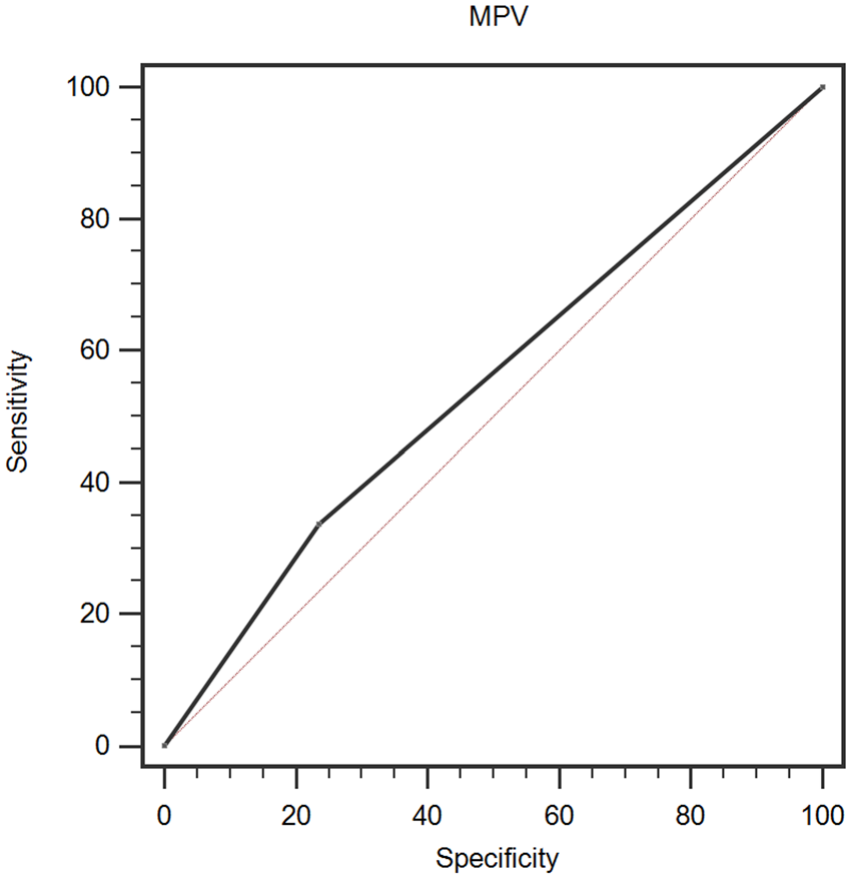
Optimized cut-off was determined for MPV using standard ROC curve analysis.

A ROC curve for OS prediction was plotted to verify the optimal cut-off value for MPV, which was 8.6 (Fig. 1). It demonstrated that MPV predicts cancer prognosis with a sensitivity of 33.8% and a specificity of 76.4% (AUC = 0.551, 95% CI: 0.507–0.551, p = 0.011). Then, patients were divided into 2 groups: patients with MPV ≤ 8.6 fL and patients with MPV > 8.6 fL. There were 359 (70.5%) patients with MPV ≤ 8.6 fL and 150 (29.5%) patients with MPV > 8.6 fL.

The relationships between MPV and clinical characteristics were shown in Table 1 and Table 2. Our study revealed that MPV was associated with tumor differentiation (p < 0.001). However, no significant differences were observed between the groups with regard to age, gender, NLR, PLR, tumor location, tumor size, T classification, lymph node metastasis, distant metastasis, and clinical stage.

**Table 2:** Baseline characteristics of patients with colorectal cancer according to MPV levels.

Variables	MPV ≤ 8.6	MPV > 8.6	P value
Age (years)	58.8 (9.7)	56.6 (10.4)	0.023
Gender (male, %)	196 (54.6)	74 (49.3)	0.278
Smoker (n, %)	79 (22.0)	30 (20.0)	0.615
Drinking (n, %)	85 (23.7)	41 (27.3)	0.384
BMI (kg/m^2^)	23.4 (3.1)	23.4 (3.5)	0.822
FPG (mmol/L)	5.10 (4.65-5.77)	5.20 (4.80-5.70)	0.301
WBC (×10^9^/L)	7.05 (2.16)	6.67 (2.72)	0.096
Hemoglobin (g/dl)	128.0 (24.4)	128.9 (23.5)	0.708
Platelet count (×10^9^/L)	297.1 (95.6)	236.4 (72.3)	<0.001
PLR	2.81 (2.23)	3.02 (2.64)	0.362
NLR	179.6 (103.1)	160.0 (103.6)	0.050

Data are expressed as means (SD) or median (IQR). FPG, fasting plasma glucose; WBC, white blood cell; MPV, mean platelet volume; NLR, neutrophil-to-lymphocyte ratio; PLR, platelet-to-lymphocyte ratio. Abbreviations: see to Table 1.

**Figure 2:**
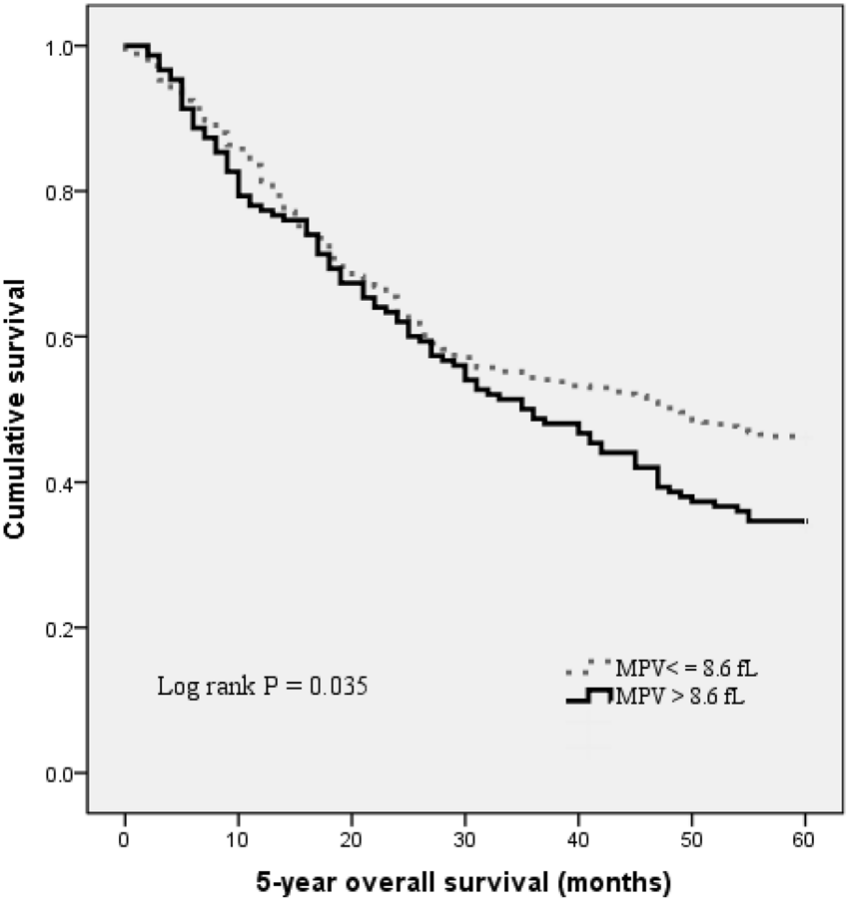
Kaplan–Meier analysis of overall survival in CRC patients.

With a median follow up of 60 months, 245 (48.1%) patients had death events. Patients with MPV ≤ 8.6 fL had a significantly better 5-year OS than patients with MPV > 8.6 fL (60.0% vs. 83.6%, p = 0.035). The Kaplan-Meier OS curves of the normal versus elevated MPV showed a significant separation (Fig. 2).

In univariate analysis, age (categorical variable), MPV (categorical variable), WBC, hemoglobin, NLR, PLR, tumor location, differentiation, T classification, lymph node metastasis, distant metastasis, and TNM stage were significant predictors of OS (Table 3). Other parameters were not found to be in correlation with OS. Next, all the factors with a P value less than 0.10 in univariate analysis were included in multivariate analysis (Table 4). In multivariate analyses, we demonstrated that MPV was an independent prognostic factor in CRC patients. Patients with MPV > 8.6 fL had a hazard ratio (HR) of 1.452 [95% confidence interval (CI): 1.118–1.884, p = 0.005] for OS.

**Table 3:** Univariate analysis of overall survival in patients with colorectal cancer.

	Hazard ratio	95% CI	*P*-value
Age (years) (≥65 versus <65)	1.891	1.484–2.409	<0.001
Gender (male versus female)	0.972	0.773–1.222	0.807
Smoker (yes versus no)	0.960	0.726–1.269	0.775
Drinking (yes versus no)	0.841	0.633–1.118	0.233
BMI (kg/m^2^)	0.981	0.945–1.018	0.315
FPG (mmol/L)	0.967	0.827–1.130	0.674
WBC (×10^9^/L)	1.116	1.068–1.167	<0.001
Hemoglobin (g/dl)	0.993	0.989–0.998	0.005
Platelet count (×10^9^/L)	1.000	0.999–1.001	1.000
MPV (fL) (>8.6 versus ≤8.6)	1.293	1.015–1.648	0.037
Tumor location			
(Colon versus Rectum)	1.383	1.100–1.739	0.006
Tumor size (cm)			
(≥5.0 versus <5.0)	1.106	0.873–1.402	0.404
Differentiation			
(Poor versus Well/moderate)	1.813	1.424–2.307	<0.001
T classification			
(T3 + T4 versus T1 + T2)	2.377	1.609–3.512	<0.001
Lymph node metastasis			
(Present versus Absent)	2.653	2.085–3.377	<0.001
Distant metastasis			
(Present versus Absent)	4.853	3.469–6.790	<0.001
TNM stage			
(III/IV versus I/II)	3.028	2.363–3.881	<0.001
PLR	1.001	1.000–1.002	0.048
NLR	1.107	1.062–1.154	<0.001

Abbreviations: see to Table 1 and Table 2.

**Table 4:** Multivariate analysis of overall survival in patients with colorectal cancer.

	Hazard ratio	95% CI	*P*-value
Age (years) (≥65 versus <65)	2.125	1.640–2.752	<0.001
WBC (×10^9^/L)	1.073	1.009–1.142	0.026
Hemoglobin (g/dl)	0.996	0.991–1.002	0.167
MPV (fL) (>8.6 versus ≤8.6)	1.452	1.118–1.884	0.005
Tumor location			
(Colon versus Rectum)	1.230	0.954–1.584	0.110
Differentiation			
(Poor versus Well/moderate)	1.446	1.129–1.851	0.003
T classification			
(T3 + T4 versus T1 + T2)	1.219	0.800–1.857	0.358
Lymph node metastasis			
(Present versus Absent)	2.278	1.102–4.706	0.026
Distant metastasis			
(Present versus Absent)	3.613	2.413–5.409	<0.001
TNM stage			
(III/IV versus I/II)	1.158	0.538–2.496	0.707
PLR	1.000	0.998–1.001	0.700
NLR	1.040	0.963–1.122	0.318

Variables that showed a p-value < 0.10 in univariate analysis were included in a multivariate Cox proportional hazards regression model. CI, confidence interval. Abbreviations: see to Table 1 and Table 2.

## Discussion

This study found that MPV is correlated with patient’s survival and is an independent risk factor for prognosis. Our findings indicted the potential importance of assessing CRC prognosis by combining clinicopathological characteristics with platelet index.

Thrombocytosis is associated with reduced survival in several cancers, such as lung cancer, ovary cancer, endometrium cancer, rectum cancer, kidney cancer, gastric cancer, pancreas cancer, and breast cancer. Increased platelets facilitate cancer progression and metastasis by promoting angiogenesis and tumor cell establishment at distant sites^15^.

The explanation for the association between MPV and survival in CRC is not fully understood. Numerous studies have identified enhanced platelet activation occurred in CRC patients. Phospholipase A2 and platelet-activating factor (PAF) acetylhydrolase were two enzymes implicated in PAF production and degradation. Previous study reported that those two enzymatic activities were elevated in CRC patients^16^. Platelet-derived endothelial cell growth factor, an angiogenic factor, was found to undergo increased expression and to be an independent prognostic factor in CRC patients^17, 18^. Moreover, permanent inactivation of platelet COX-1 by low-dose aspirin might restore anti-tumor reactivity^19^. These data are also consistent with the current knowledge that anti-platelet is considered to be a part of cancer adjuvant therapy^1^. MPV is an early indicator of activated platelets and is available in any clinical practice. Platelets with elevated MPV contain more α-granules and release more prothrombotic substances, and these substances aggravate inflammation^20^. Recent studies confirmed higher levels of MPV in CRC patients^13, 14^. The current study further revealed the prognostic role of preoperative MPV in CRC patients. After curative treatment, 30% of patients with stage I-III and up to 65% of patients with stage IV CRC develop recurrent disease^21^. Our previous study showed the patients with colon cancer had higher MPV levels compared with normal controls, and MPV levels were reduced after surgery^13^. Therefore, MPV may be an easy approach for surveillance in post-operative CRC patients. In addition, MPV could be assessed in routine clinical practice without extra cost.

The potential limitations of the present study should be acknowledged. First, this was a single-center retrospective study and additional larger validation studies are needed to confirm our results. Second, we were unable to explore the exact mechanism of MPV in CRC. Third, the patients were composed of Chinese. The application to other ethnic groups still needs further investigation.

In conclusion, MPV is easily available with routine blood counts. Elevated MPV may serve as a marker of adverse prognosis in CRC. Further studies are warranted to clarify the exact role of MPV in CRC.

## Patients and Methods

### Study population

The records of 509 consecutive CRC cases who were admitted to Harbin Medical University Cancer Hospital, Harbin Medical University between January 2009 and December 2009 were retrospectively reviewed. All patients undergone surgical resection. The pathologic diagnoses of CRC were evaluated by two pathologists from biopsy reports. All cases were categorized according to the WHO classification. The pathologists reviewed all biopsy slides and scored the pathologic variables into 3 classes according to the WHO classification as follows: well differentiated (tubular appearance and mild/moderate atypia), moderately differentiated (lumen formation with moderate/severe atypia) and poorly differentiated (solid or medullary appearance, with severe dysplasia). None of the patients received preoperative chemotherapy or radiation therapy. After surgery, patients were treated with radiotherapy and/or chemotherapy or not. 71 patients who were at high risk of local recurrence and distant metastasis due to rejection, poor physical condition or side effects, did not receive adjuvant therapy. Patients were excluded if they had hematological disorders, coronary artery disease, hypertension, diabetes mellitus, and medical treatment with anticoagulant, statins, and acetylic salicylic acid.

Standard demographic and clinicopathological data were collected from the patients’ records in hospital. Survival data were obtained through follow-up. Overall survival (OS) was defined as the interval from the date of diagnosis to death or last follow-up. The median follow-up time was 60 months.

The Institutional Ethics Review Board of Harbin Medical University Cancer Hospital approved this study prior to commencement of data collection and waived the informed consent requirement because it was a retrospective study. All studies were conducted according to guidelines (Declaration of Helsinki) for biomedical research.

### Biochemical measurements

Venous blood samples after an 10-hour overnight fasting were collected from the individuals within 1 week prior to surgery. White blood cell (WBC), haemoglobin, and platelet indices were measured by an autoanalyzer (Sysmex XE-2100, Kobe, Japan). The whole blood samples were collected in EDTA-containing tubes, and all samples were processed within 30 minutes after blood collection.

### Statistical analysis

The descriptive statistics are presented as means ± SD or medians (interquartile range) for continuous variables and percentages of the number for categorical variables. Inter-group differences in categorical variables were assessed for significance using the Chi-square test; differences in continuous variables were assessed using the Mann-Whitney U test or t-test. The platelet-to-lymphocyte ratio (PLR) was calculated as the absolute platelet count measured in ×10^9^/L divided by the absolute lymphocyte count measured in ×10^9^/L. The neutrophil-to-lymphocyte ratio (NLR) was calculated as the absolute neutrophil count measured in ×10^9^/L divided by the absolute lymphocyte count measured in ×10^9^/L. Kaplan-Meier analysis was used to assess survival. Log-rank tests were used to compare survival of patients between subgroups. The Cox proportional hazards regression model was applied to evaluate multivariate analyses, and those statistically significant characteristics in univariate analysis (p value <0.1) were used to perform multivariate analysis. ROC analysis was conducted to select the optimum cut-off value of MPV dichotomize the patients into low and high groups. A value of p < 0.05 was regarded as a significant difference between groups. All statistical analyses were performed using SPSS Statistics version 22.0 (SPSS Inc., Chicago, IL, USA).
